# Cryopreservation and culture strategies for testicular tissue and cells in small and large animals

**DOI:** 10.3389/fvets.2025.1638248

**Published:** 2025-07-16

**Authors:** Valeria Vurchio, Martina Colombo, Rolando Pasquariello, Gaia Cecilia Luvoni

**Affiliations:** ^1^Department of Veterinary Medicine and Animal Sciences (DIVAS), Università degli Studi di Milano, Milan, Italy; ^2^Department of Agricultural and Environmental Sciences - Production, Landscape, Agroenergy, Università degli Studi di Milano, Milan, Italy

**Keywords:** animals, freezing, *in vitro* spermatogenesis, stem cells, testicular tissue, vitrification

## Abstract

The preservation of testicular tissue and male germ cells represents a cutting-edge technique for safeguarding fertility, especially when sperm collection is not possible, such as in prepubertal animals, those that die unexpectedly or that receive gonadotoxic therapies after cancer detection, and in adult males suffering from some pathology related to azoospermia. Current methods under investigation include the optimization of cryopreservation protocols, as well as the development of culture platforms to enable *in vitro* spermatogenesis (IVS). Although these approaches are still in the research and development phase, they have shown promising potential for male fertility preservation. Cryopreservation is a common method for long-term *in vitro* storage of tissue and cells, which enables the maintenance of reproductive capacity across different animal species and contributes to the creation of gene banks for endangered species. Spermatogenic cells from cryopreserved testicular tissue can be cultured *in vitro* and resume their functions after thawing, contributing to the preservation of fertility and genetic resources in both small and large animals. The main challenges of IVS include providing a suitable microenvironment that mimics the testicular niche to support the survival and development of all the cell types, as well as to achieve complete differentiation toward spermatozoa. Therefore, there is a great interest in developing methods to study IVS, both for basic research and clinical application. Given the importance of this topic, this review aims to provide an overview of recent advancements in the cryopreservation and culture of both testicular tissue and cells for preserving male fertility in large and small domestic animals.

## 1 Introduction

Over the past few decades, significant advancements have been made in both cryopreservation and *in vitro* culture strategies aimed at preserving testicular tissues and maintaining the viability and functionality of spermatogonial stem cells (SSCs) across various animal species (1–3). This progress holds great promise for long-term fertility preservation and genetic conservation, particularly in prepubertal individuals or valuable breeding animals unable to produce mature sperm (1). However, the direct application of these technologies across different animal species remains challenging, often requiring protocol adjustments and further refinement (1). Therefore, this review aims to summarize and critically assess current cryopreservation and culture strategies for testicular tissues and cells across different animal models, discussing the methodologies, challenges, and progress in maintaining cellular integrity and promoting spermatogenic potential. By exploring these advancements in both small and large animals, this review highlights the current state of the art and outlines opportunities for further refinement and application. The manuscript is structured in two main sections: the first part provides an overview of cryopreservation techniques for testicular tissues and cells, including their principles, applications, and devices used; the second part focuses on *in vitro* culture systems, discussing their design, outcomes, and potential to support spermatogenic progression.

## 2 Cryopreservation strategies for testicular tissues and cells

Methods and protocols for storing male germplasms have been developed over the years to preserve fertility, promote the spread of specific genotypes and protect biodiversity in endangered breeds or species (4). Semen freezing is a widely recognized method for genome conservation and is commonly applied in infertility laboratories (5) but this method cannot be used for juvenile and pre-pubertal individuals whose gonads have not started producing spermatozoa. When semen preservation is not feasible, cryopreservation of testicular tissue fragments or testicular cell suspensions, containing early/immature germ cells, may potentially expand the range of biotechnological applications for germplasm and fertility preservation (6). These strategies have been applied in several animal models (7–15) and, even if the protocols are still experimental, they are very promising for application in assisted reproductive technologies (ARTs) (4). Testicular tissue cryopreservation enables the preservation of SSCs while maintaining critical cell–cell interactions and structural integrity, thus facilitating the restoration of gametogenesis through transplantation or IVS (16). This approach has been investigated in numerous studies focusing on the collection and preservation of testicular tissue from both sexually immature individuals (12, 17, 18) and adult animals (8, 19). However, testicular tissue is composed of various cell types, including germ cells, Sertoli cells, and Leydig cells, which work together to support gamete production and hormone secretion (20). Due to its complexity and cellular heterogeneity, testicular tissue cryopreservation is more challenging than preserving SSCs alone, as it requires maintaining intercellular interactions and ensuring post-thaw viability to restore spermatogenesis *in vitro* (6). Spermatogonial stem cells are responsible for the continuous production of spermatozoa through self-renewal and differentiation (21). Their cryopreservation, although technically demanding and still experimental, has been explored as an alternative strategy to tissue preservation, offering more control over cryopreservation parameters due to the cellular homogeneity (22). However, despite the development of different protocols in several animal species including goat (23), cattle (24) and horses (5), there are still no documented live birth resulting from cryopreserved SSCs in large animals (6).

The two most widely used cryopreservation methods are slow freezing and vitrification. Few reports used a modified technique called rapid freezing (Table 1) (5, 25, 26).

**Table 1:** Cryopreservation strategies for testicular tissues and cells.

Method	Cooling rate	CPAs used	Advantages	Disadvantages	Example of application (species)
Slow freezing	Controlled slow rate (~ − 1°C/min)	Low concentration (e.g., DMSO, glycerol, ethylene glycol)	Low CPA toxicity; minimal technical training needed; low contamination risk (closed system)	Risk of ice crystal formation; requires expensive equipment; time-consuming	Canine (63), Feline (62), Ovine (7), Porcine (9), Bovine (14)
Rapid freezing	Intermediate (vapor-phase cooling)	Moderate to high concentration (e.g., DMSO, glycerol, ethylene glycol)	Faster than slow freezing; reduced equipment requirements	Risk of intracellular ice formation; less standardized protocols	Equine (5), Feline (55)
Vitrification	Ultra-rapid (direct plunge into LN₂)	High concentration (e.g., DMSO, glycerol, ethylene glycol)	Fast procedure; avoids ice formation; low cost	High CPA toxicity; technically demanding; risk of contamination (open systems)	Canine (17), Feline (12), Ovine (77), Porcine (48), Bovine (66)

CPA, cryoprotectant; DMSO, dimethyl sulfoxide; LN₂, liquid nitrogen.

Slow freezing, controlled using programmable freezers, involves the use of low concentrations of permeable cryoprotectants (CPAs) and gradual cooling (27). This approach allows for progressive cell dehydration and minimizes intracellular ice formation before storage in liquid nitrogen (1). However, it also carries a significant risk of freeze-induced injury and it is a time-consuming and costly process, as it requires expensive equipment (28).

Rapid freezing, or vapor fast freezing (VFF), involves sequential treatment with higher concentrations of CPAs, followed by exposure to liquid nitrogen vapors before immersion into liquid nitrogen (6, 28, 29). One of the main challenges of this technique is the risk of cryoinjury caused by intracellular ice formation, which can compromise cell viability (26).

Vitrification involves the transformation of water or water-based solutions into an amorphous, glass-like vitreous state without the formation of ice crystals (30). This method is generally faster than slow freezing and significantly reduces the formation of both intracellular and extracellular ice by utilizing high concentrations of CPAs and ultra-rapid cooling rates (28). To counteract CPAs toxicity, a preliminary equilibration with lower CPA levels is typically performed before exposing cells to the final vitrification solution (6).

One of the key advantages of vitrification is the minimal risk of freezing injury, resulting in a higher cell survival rate. However, this technique requires a high level of technical expertise (31). Moreover, despite its cost-effectiveness, the high CPAs concentrations necessary for vitrification may compromise the morphological and functional integrity of cells (32).

Given the complexity of preserving testicular tissue and cells, further optimization of processing and storage methods remains essential to improve cryopreservation outcomes (1, 30, 33).

### 2.1 Sample preparation

To provide optimal cryoprotection, CPAs must effectively diffuse in and out of the tissue to minimize cryoinjury. Therefore, the size of testicular tissue samples should be carefully considered (34). Particularly for vitrification, sample size plays a pivotal role in determining the likelihood of successful solidification of the aqueous environment of tissue and cells into a non-crystalline, glass-like state. Additionally, sample size has a role in the prevention of devitrification, which occurs during warming and is characterized by the formation of ice crystals (35).

Testicular tissue sizes ranging from 0.3 to 1.5 mm^3^ are commonly used in cryopreservation across various animal species (36–39). Among testicular cells, SSCs are a rare subset of germ cells, representing only 0.2–0.3% of the germ cell population in bovine species (40). However, the proportion of SSCs in the testes of other livestock species, such as sheep, goats, pigs, and buffalo, remains undocumented (21). In the tissue, this heterogeneous cell population varies in function, size, water content, and membrane permeability, and the scarcity of SSCs further complicates the development of an efficient cryopreservation protocol (1).

The stem cells are typically isolated from testicular tissue through enzymatic digestion or mechanical isolation. Enzymatic digestion, using enzymes such as collagenase IV, trypsin, DNase I, and hyaluronidase, is commonly employed to dissociate testicular cells. Since no single enzyme is sufficient to isolate SSCs effectively, multi-step or sequential enzymatic digestion protocols are often used (41). The mechanical isolation method involves the removal of the tunica albuginea and visible connective tissues, followed by mechanical dissociation of seminiferous tubules using scissors and forceps, with subsequent filtration of the cells (42).

While enzymatic digestion generally yields higher numbers of SSCs compared to mechanical dissociation (43), both these methods may negatively affect the viability and functionality of germ cells, as well as alter their biophysical properties, leading to increased cellular sensitivity to freezing processes. Moreover, the disruption of cell-to-cell interactions may negatively affect cell proliferation and differentiation (44). In contrast, preserving testicular tissue maintains the *in situ* structure and cellular relationships, including the spatial arrangement of somatic cells and germ cells, which are essential for studying spermatogenesis and testicular function (43).

### 2.2 Cryopreservation carriers

The device used for supporting the cryopreservation of samples can be an additional factor influencing the outcome of the process. While freezing of testicular tissue and cells is generally performed in cryovials or straws (45), studies on vitrification carefully select the appropriate vessel for the procedure, although plastic straws are also used in conventional vitrification due to their practicality, low cost, and space optimization within cryogenic cylinders (46). Vitrification methods can be classified as either “open vitrification” or “closed vitrification.” Open devices allow direct contact of the sample with liquid nitrogen for faster heat transfer (6). However, this direct contact introduces the risk of pathogen transmission to the sample during cooling and increases the potential for cross-contamination within the container (1). In contrast, closed systems prevent direct contact between the sample and the cooling solution during freezing or storage, thus addressing the contamination issue. A limitation of closed systems is that they result in slower cooling rates, requiring higher concentrations of CPAs to prevent ice crystal formation. This, in turn, increases the potential cytotoxicity of the CPAs, making the protocols more hazardous for cells (47). A summary of the main types of carriers used in cryopreservation, along with their characteristics, is provided in Table 2.

**Table 2:** Cryopreservation carriers for testicular tissues and cells.

System type	Examples of cryo-containers	Contamination risk	Technical complexity	Usage areas
Open	SSV, NIV, Cryotop	High	Moderate to high: requires precise handling and rapid timing	Testicular tissue (9, 19, 58) and cells (77)
Closed	Plastic straws, Cryotubes/Cryovials, E. Vit device	Low	Low to moderate: easier to handle	Testicular tissue (38, 46, 52) and cells (24, 74)

SSV, solid surface vitrification; NIV, needle immersed vitrification.

A simple and rapid vitrification method for testicular tissue has been reported in the ovine model using a novel device called *E. Vit* (FertileSafe, Ness Ziona, Israel), which enables all cryopreservation procedures to be performed within a straw. The device consists of a 0.3 mL straw with a 50 μm pore polycarbonate grid at one end, facilitating ultrarapid vitrification of tissues and cells with minimal volume. This design allows for the expulsion of excess CPAs while preventing sample loss (38). Using this device, ovine pre-pubertal testicular tissue (1 mm^3^) maintained plasma membrane integrity at 66.00% immediately after warming and 59.67% after 2 h of *in vitro* culture (IVC). However, extended culture up to 24 h post-warming led to a significant decrease in membrane integrity to 31.00%, and a stress response was observed (38).

Carrier-free systems, such as Solid Surface Vitrification (SSV), offer an alternative approach. Introduced in 2010, SSV is a containerless vitrification method that enables open vitrification of tissues and cells (36). The procedure involves immersing testicular tissue samples in a vitrification solution before placing them on a sterile aluminum boat floating on liquid nitrogen, then transferring them into precooled vials and submerging them in cryogenic storage (36). This method has proven to be an efficient method for vitrifying testicular biopsies in porcine models (36, 48, 49) and prepubertal domestic cats (12).

### 2.3 Warming rates

Whether freezing is allowed (as in conventional cryopreservation) or prevented (as in vitrification), the CPAs that have penetrated the internal compartments of a multicellular system must diffuse back through several membranes within the tissue during warming, with each membrane acting as a barrier (34).

Intracellular ice formation is considered the primary cause of cell damage induced by cryopreservation, even during the thawing process, when recrystallization may occur. Recrystallization refers to the growth of ice crystals, starting from small ice nuclei, into fully-fledged intra- and/or extracellular ice crystals, which increase in size as the temperature rises. During this process, cells must return to their original isotonic conditions, and uncontrolled water influx into the cells can generate osmotic stress and cellular swelling, leading to damage to the plasma membrane and subsequent cell lysis (27). To minimize osmotic shock, it is important to use a set of media with gradual decrease of the osmotic pressure (35).

Therefore, tissue and cell survival after freezing relies on effective thawing and CPAs removal to preserve integrity and minimize damage (50).

However, research addressing these issues has been limited. Tissues and cells are typically removed from storage by rapid warming followed by gradual removal of CPAs. The temperature during warming can significantly impact the outcome. For instance, thawing adult bovine tissue at 37°C and 97–100°C has been shown to result in better cell viability and spermatogonial cell survival compared to thawing at 4°C (51). Additionally, Lima et al. (52) demonstrated that warming at 50°C for 5 s can effectively ensure the reanimation and survival of vitrified testicular tissues from prepubertal domestic cats, but additional research is required to better understand the impact of warming protocols on avoiding devitrification and ice recrystallization and ensuring the optimal revival of tissue and cells (52).

## 3 Cryopreservation of testicular tissue and SSCs

### 3.1 Small animals

Relevant advancements in testicular tissue cryopreservation have been reported in small animal species, particularly cats and dogs, although at varying levels of experimental validation. The most significant achievement in this context was reported in the domestic cat, where spermatozoa retrieved from frozen–thawed testicular tissue were used for intracytoplasmic sperm injection (ICSI), resulting in the birth of live kittens after embryo transfer (53). Although this strategy did not involve grafting or IVS, it provided clear proof of functional sperm recovery leading to viable offspring. In contrast, earlier attempts involving xenografting of cryopreserved cat testicular tissue into immunodeficient mice did not yield successful preservation of germ cells (54), highlighting the challenges of this approach for restoring spermatogenesis in this species.

Comparative evaluations of cryopreservation techniques have further refined experimental protocols. In cats, rapid freezing - typically preceded by a pre-equilibration phase at 4–5°C - has consistently shown better preservation of sperm plasma membrane integrity and seminiferous epithelium compared to slow freezing (26, 55), while vitrification has emerged as a promising alternative, with several studies reporting good structural maintenance and reduced interstitial damage, indicating that ultra-rapid cooling may represent a valid alternative to conventional protocols (12, 19, 46, 56, 57). In dogs, needle-immersed vitrification (NIV) has been introduced to facilitate handling of small tissue fragments. Using NIV, higher preservation of undifferentiated germ cells was observed compared to slow freezing (58). However, findings in gray wolves (*Canis lupus*), a species closely related to domestic dogs, indicate species-specific variability, as slow freezing proved more effective than NIV (11).

The choice of CPAs also plays a critical role and appears to be species-specific. The combination of dimethyl sulfoxide (DMSO) and glycerol proved to be the most effective in the cat, ensuring superior preservation of the seminiferous epithelium and greater proliferative potential in both freezing and vitrification protocols (12, 37, 52, 56, 57). These compounds consistently maintained tubular architecture and cellular viability better than alternatives like ethylene glycol, which was associated with increased cytotoxicity and morphological disruption in several feline studies (46, 56). Conversely, in canine testicular tissue, favorable results were reported using DMSO in combination with EG in both pre- (17) and post- pubertal (58, 59) specimens, with preserved nuclear and tissue architecture despite some mild alterations like basement membrane detachment.

In cats, where more studies are available, tissue fragment size and warming rates have also been investigated. Larger tissue fragments (0.5 cm^3^) cryopreserved with glycerol showed better morphological features, although subsequent assessments of apoptosis and DNA integrity did not reveal significant differences between fragment sizes (37, 60). In prepubertal animals, exposure to 50°C for 5 s consistently resulted in better preservation of seminiferous tubule structure and enhanced somatic and germ cell viability compared to standard warming at 37°C (52) or higher temperatures such as 60°C (57). These results indicate the importance of integrating morphological and molecular assessments in protocol optimization and underscore the importance of fine-tuning warming conditions to maximize recovery after vitrification.

Cryopreservation of testicular cell suspensions, although still limited to unsorted populations, has shown promising results in both cats and dogs. In felines, slow freezing with 7.5% DMSO yielded the highest recovery rates (61), and one study reported that cell suspensions may better preserve sperm membrane integrity than tissue fragments (62). In canines, while SSC-specific cryopreservation protocols have not yet been developed, testicular cell suspensions have demonstrated the ability to colonize recipient testes after xenotransplantation into immunodeficient mice. However, no differentiation has been observed, likely due to the evolutionary distance between donor and recipient species (63, 64).

### 3.2 Large animals

In large animals, the efficacy of cryopreservation techniques for testicular tissue has shown species-specific trends.

In ovine species, slow freezing has proven more effective than vitrification in preserving immature testicular tissue integrity and functionality. These findings are supported by both *in vivo* data, demonstrating that slow freezing maintains seminiferous tubule architecture and spermatogenic activity after xenografting (7), and *in vitro* findings, which confirm better preservation of morphological features, extracellular matrix components, and gene expression profiles following cryopreservation by slow freezing, especially with 5 mm^3^ tissue fragments (18).

In porcine species, both slow freezing and vitrification have yielded comparable outcomes in terms of germ cell survival and DNA integrity, with several studies reporting no significant differences between the two methods (9, 36, 58). Notably, Kaneko et al. (48) demonstrated that vitrified immature testicular tissue retained functional germ cells, as evidenced by the birth of live piglets following xenografting.

In cattle, slow freezing is the most widely adopted method and has consistently provided reliable preservation of tissue structure and cell viability. Zhao et al. (65), for instance, reported that slow-frozen calf testicular tissues retained structural integrity and functional potential after xenotransplantation, with preserved seminiferous cords, angiogenesis, and increased expression of germline and somatic markers. However, a recent comparative study showed that vitrification, although associated with lower attachment of seminiferous tubules to the basement membrane, preserved germ and Sertoli cells, maintained membrane integrity, and reduced apoptosis—supporting germ cell viability and colony formation in short-term *in vitro* culture (66).

Among the various CPAs, DMSO remains the most effective for preserving testicular tissue structure and germ cell functionality across species. In ovine and equine models, DMSO-based slow freezing protocols have led to superior outcomes, including better seminiferous tubule integrity, reduced basement membrane disruption, and preserved germ cell viability and SSC marker expression (7, 67). When directly compared to other CPAs such as ethylene glycol, propylene glycol and glycerol, DMSO has also shown greater efficacy in porcine (48) and bovine (51) models. Notably, DMSO performance remained consistent regardless of animal tissue age (immature vs. adult) and cryopreservation strategy (slow freezing vs. vitrification), often outperforming alternative CPAs in preserving both cellular integrity and molecular functionality (36, 68–71). Additionally, recent studies suggest that DMSO may support DNA repair mechanisms post-thaw (71).

Cryopreservation outcomes have been further improved by supplementing DMSO-based media with protective additives. Knockout serum replacement (KSR), for example, has proven to be a valid alternative to fetal bovine serum (FBS), ensuring consistent cryoprotection and gonocyte recovery in both immature and adult bovine tissues (8, 39). Trehalose has also emerged as a particularly effective additive, showing consistent benefits across species. Its inclusion in cryopreservation protocols has been associated with improved antioxidant activity, enhanced cell viability and reduced apoptosis, supporting both the structural integrity and functional capacity of SSC-containing germ cells (8, 72, 73). Notably, trehalose-based vitrification strategies have also yielded promising results in the porcine model. In particular, the inclusion of trehalose in the vitrification medium was associated with preserved tissue viability after warming and, remarkably, enabled the generation of viable offspring from sperm retrieved from xenografted tissue, highlighting the long-term potential of trehalose in supporting germline functionality following cryopreservation (48).

The post-thaw recovery and transplantation efficiency have been reported to be higher when cryopreserving testicular tissue compared to isolated cells in both bovine and porcine species (15, 24). Nevertheless, the cryopreservation of isolated SSCs remains a promising strategy. In large animal species, slow freezing remains the most widely used technique for SSC cryopreservation, providing consistent results in terms of post-thaw viability and proliferative capacity. Studies conducted in sheep (74), cattle (14), pigs (75) and horses (5) have demonstrated that SSC-enriched suspensions preserved via slow freezing maintain cellular integrity and are capable of surviving and proliferating after thawing. Supporting this, Oatley et al. (76) showed that bovine SSCs cryopreserved using a simple slow freezing protocol retained their functional potential, as evidenced by their colonization of recipient mouse seminiferous tubules following transplantation.

Although vitrification is still underexplored in this context, promising results have been reported by Patra et al. (77) who showed that vitrified goat SSCs retained post-warming viability and colony-forming capacity, despite signs of oxidative stress and partial mitochondrial dysfunction. These findings suggest that vitrification may offer a viable alternative to slow freezing, though further refinement is needed to improve its consistency and efficacy.

As in testicular tissue, the choice and combination of CPAs plays a central role in SSC cryopreservation outcomes. Dimethyl sulfoxide remains the most widely used CPA across species, often combined with non-permeating agents such as sucrose or trehalose to enhance membrane protection during freezing and enhance post thaw outcomes. Consistent with findings in tissue cryopreservation, trehalose has been shown to enhance the viability, recovery, and proliferative capacity of SSCs in ovine (78), porcine (15), and bovine (24) models, with cryopreserved cells also demonstrating colony-forming potential after xenotransplantation. Similarly, sucrose has proven effective as an osmotic buffer and membrane stabilizer, with improved survival and proliferation of SSCs observed in sheep (79), cattle (14) and pigs (75) when added to DMSO-based media.

Beyond sugars, other strategies have aimed to mitigate oxidative damage associated with cryopreservation. In goats, the addition of melatonin (10^−6^ M) to the freezing medium improved mitochondrial function, antioxidant capacity, and overall cell viability while reducing apoptosis and autophagic activity (80). In cattle, a synergistic effect between DMSO and propanediol was also reported, leading to higher post-thaw viability and membrane integrity than either CPA alone (81). The robustness of DMSO-based protocols is also confirmed in equine species, where cryopreserved SSCs retained viability, metabolic activity, and expression of key stem cell markers (5).

Altogether, these findings highlight the need for species-specific and application-oriented protocols that consider the structural complexity of testicular tissue and the inherent sensitivity of SSCs, thereby supporting the development of effective fertility cryopreservation strategies both in small and large animals.

## 4 Recreation of spermatogenesis *in vitro*

Although cryopreservation is an effective strategy for fertility preservation, its combination with transplantation or *in vitro* culture techniques is essential to develop mature germ cells and obtain progeny. The culture of testicular cells, fresh or preserved, is aimed at achieving these results *in vitro*, without the use of experimental animals. In addition, recreating spermatogenesis *in vitro* in animal species holds significant importance for understanding reproductive biology, preserving endangered species, and advancing biotechnological applications such as ARTs and genetic conservation.

However, faithfully recreating the entire process *in vitro* remains a challenge. The main obstacles include the possibility to provide a suitable microenvironment that mimics the testicular niche to support the survival and development of all the cell types, as well as to achieve complete and functional spermatogenesis. In domestic species, several IVS approaches have been explored for propagation and differentiation of spermatogonia *in vitro* into mature sperm. These include the culture of testicular tissue explants, isolation and culture of SSCs and generation of three-dimensional (3D) culture platforms. Among these, the culture of SSCs using 3D culture models, such as organoids and decellularized tissue, is garnering increasing interest.

In this section, we will summarize the strategies developed for culturing testicular tissue and cells with the goal of recreating IVS in large and small animals (Table 3). Due to the limited literature available for both, the section is organized by techniques rather than by species, discussing the results across species when relevant.

**Table 3:** In vitro spermatogenesis approaches across species.

Culture system/ Method	Species	Culture duration	Main outcomes	Functional results	References
Testicular fragments cultured using air liquid interface on 0.45 μm pore membranes	Cattle	2 weeks	Low maintenance of seminiferous tubules and increase in germ cell nuclei	SSC proliferation without meiosis and differentiation	(82)
Testicular fragment cultured using air liquid interface (3D) using agarose gel blocks or encapsulated (2D) into agarose gel	Sheep	48 h	Moderate morphological alterations of seminiferous tubules	Structural protection of the tissue and SSC preservation	(83)
Testis organ culture using air liquid interface using agarose gel blocks	Cat	6 weeks	Low alteration of tissue morphology and low preservation of spermatogonia	Maintenance of tissue morphology; no germ cell development and differentiation	(84)
In vitro culture of SSCs	Goat, Pig, Buffalo, Cattle	From 2 weeks to 2 months depending on the species	SSC self- renewal and propagation	SSC propagation without differentiation	(41, 89–91, 94)
SSC in vitro culture and transplantation	Dog	2 weeks	SSC self-renewal, propagation and differentiation	Full spermatogenesis, resulting in epididymal sperm	(102)
Testicular organoids	Cattle	21 days	Organoids containing Leydig, Sertoli, and peritubular myoid cells	Steroidogenic activity, but no spermatogenesis in vitro due to the absence of spermatogonia	(107)
Testicular organoids	Cattle	28 days	Organoids containing Sertoli cells and gonocytes	Establishment of gonocyte organoid and their progression toward SSCs	(108)
Organoids cultured on testis-derived decellularized scaffold	Pig	45 days	Development of organoids containing Sertoli cells, Leydig and germ cells assembled	Establishment of organoids characterized by seminiferous tubules-like structures, but no germ cell maturation achieved	(115)
Organoids cultured on testis-derived decellularized scaffold	Ram	30 days	Development of organoids containing neonatal testicular cells	Steroidogenic activity and differentiation of spermatogonia in post meiotic cells	(116)

SSCs, spermatogonial stem cells.

### 4.1 Testicular tissue explants

Testicular tissue fragments can be readily isolated from both immature and mature animals, both large and small, and cultured under controlled conditions to preserve cellular viability, proliferation, and differentiation. This *in vitro* culture aims to replicate the testicular microenvironment, which is essential for supporting spermatogenesis. *In vivo*, germ cells, Sertoli cells, peritubular cells, interstitial cells, and particularly Leydig cells must maintain normal morphology to synthesize autocrine and paracrine factors vital for spermatogenesis. Therefore, it is crucial that *in vitro* cultures preserve the testicular structure to maintain paracrine signaling, which is essential for germ cell proliferation and differentiation. Various culture systems for testicular tissue fragments from different species have been reported to enhance the stabilization of these fragments. An early study in cattle demonstrated the survival and proliferation of bovine SSCs during a two-week explant culture, where tissue fragments were cultured on the top of 0.45-mm pore membranes to create an air liquid interface culture system (82). Morphological analysis revealed maintenance of seminiferous tubular structure and a significant increase in germ cell nuclei per tubule compared to fresh tissue (82). However, no meiotic cells were observed, indicating spermatogonial proliferation without further differentiation (82). Despite these promising results, careful monitoring of the culture medium volume in each well is crucial when using transwell insert membranes. The medium level should be sufficient to contact the bottom of the insert without submerging the tissue. Hypoxia also limits the efficiency of organ culture. Recently, in sheep, culturing testicular fragments in agarose gel resulted in less cell loss and basement membrane disruption, suggesting structural protection of the tissue (83). This 3D system, however, may impede medium perfusion due to the rigidity of the agarose gel, which can affect both tissue architecture and function.

In domestic cats, testis organ culture does not progress as observed in other mammals. While some spermatogonia, potentially including SSCs, are maintained over extended periods, there is no advancement in germ cell development (84). This issue pertains not only to the initiation of spermatogenesis but also to its progression; even tissues containing more developed germ cells at the outset show no further differentiation. The complexity of spermatogenesis initiation and regulation in domestic cats appears to be greater than in other mammal species such as bull, buffalo, ram, goat, boar, wild boar, dog and rabbit (85). In particular, the overall rate of spermatogenesis in cats is lower compared to several other mammals, as indicated by a lower meiotic index (84). Additionally, abnormalities in the seminiferous epithelium are commonly observed in cats (84).

Most of these studies have been based on short-term culture time which is a limit if it is considered that spermatogenesis takes place in about 3 months in many species. Recently, it was demonstrated that small intact testicular tissue fragments cultured in knockout serum replacement could be effectively maintained *in vitro* for up to 4 weeks of culture (86). Testicular tissue integrity was dependent on fragment size and preparation method, where the smallest size and intact preparation method were advantageous.

Taken together, these findings indicate that for this type of culture platform culture time can be affected by several factors including tissue fragment size, preparation method, media supplements, matrix and serum sources (86). It is therefore important to consider all these factors to establish a long-term *in vitro* maintenance of the testicular tissue.

### 4.2 Culture and differentiation of spermatogonia *in vitro*

*In vitro* culture of spermatogonia, particularly SSCs, is essential for self-renewal, differentiation, and manipulation of testicular germ cells. Various culture systems and medium compositions have been developed to enhance SSC viability and proliferation (87, 88). However, long-term SSC cultures exceeding 2 months have not been established yet for domestic animals, with current efforts limited to short-term cultures in species such as goat (41), pig (89), buffalo (90) and cattle (91). To date, complete *ex vivo* spermatogenesis has been achieved in the murine models (92, 93), while, in domestic species, propagation of spermatogonia without effective meiotic division has only been reported.

Regarding media composition, two types, stempro-34 and DMEM supplemented with Fetal Bovine Serum (DMEM-FBS), have been utilized for SSC culture in domestic animals. Colony formation has been observed in SSCs culture of goats and pigs using DMEM-FBS medium, with these colonies containing PGP9.5-positive cells, a marker of undifferentiated spermatogonia, including SSCs (89, 94). Similarly, colonies formed in SSC cultures of piglets and calves using serum free stempro-34 medium contained *Dolichos biflorus* agglutinin (DBA)-positive cells (95, 96). In previous studies, spherical cell colonies (SDC) have been observed in porcine testicular cell culture containing PGP9.5-positive cells with stem and germ cell characteristics (97). Additionally, growth factors are essential to form the SDC in SSC cultures. In pig SSC cultures, epidermal growth factor (EGF) and fibroblast growth factor (FGF) positively influenced the number and size of SSC-like colonies, and their addition to primary cell cultures of neonatal pig testes influenced NANOG, PLZF, OCT4, and GATA4 transcript level (95). Furthermore, FGF2 has been shown to mediate mouse SSC self-renewal via up- regulation of Etv5 and Bcl6b through MAP2K1 activation (98). These findings suggest that SSC colonies can be formed in both stempro-34 and DMEM-FBS media and that FGF plays a significant role in SSC cultures. More recently, in dogs, colonies were observed in both media at day 7, and the addition of FGF significantly affected colony formation from two-month-old beagle’s testes (99). These results indicate that stempro-34 and DMEM-FBS media, supplemented with glial cell line-derived neurotrophic factor (GDNF) and FGF are well suited for deriving SDCs from neonatal beagle testes.

The use of DMEM-FBS and stempro-34 has not been the only approach used to culture spermatogonia. In cattle, spermatogonia were successfully cultured from cryopreserved testicular tissues using 2i medium (100). The obtained culture system resulted in enhanced proliferation, survival, anti- differentiation and apoptosis. These effects might be due to the attenuation of Suv39h1/2-mediated H3K9me3 level by 2i stimulation through MEK and GSK pathways (100).

In both small and large animals, advances have been made in SSC culture, demonstrating the potential of these cells to restore and produce germ cells *in vitro* (101). In dogs, SSCs were able to progress *in vivo* toward more differentiated testicular cell stages, including spermatocytes, spermatids, and sperm, following transplantation (102). Moreover, the *in vitro* generation of embryonic germ cell-like cells has also been reported in canines (101, 103). The canine model, in particular, offers promising opportunities to discover new signaling molecules, transcription factors, and mechanisms involved in self-renewal and differentiation processes critical for germ cell development.

On the other hand, in cats, Powell and colleagues (104) successfully isolated SSCs; however, no culture protocols have been developed so far. In the same study, they reported that markers commonly used for SSC identification in other species may be less reliable for isolating cat SSCs, whereas pluripotent markers, particularly SSEA-4, may provide more enriched SSC populations. SSCs are low in number in the testis, and the smallest subpopulation of spermatogonial cells identified was SSEA-4þ positive, expressing NANOG, POU5F1, and SOX2 (104).

Although several protocols to isolate and culture spermatogonia and SSCs have been developed in few domestic species, it is important to note that these culture systems consisted of multiple cell types. These included not only spermatogonia but also testicular somatic cells, such as Sertoli cells, suggesting that testicular cells rely on cell-to-cell interactions for establishing a reliable culture system that could restore spermatogenesis *in vitro*.

Last but not least, SSCs from domestic animals have been transplanted into the seminiferous tubules of germ cell-depleted infertile mice, although few of these experiments were able to restore spermatogenesis (63, 105, 106).

Overall results indicate that successful xenotransplantation is possible between SSCs and mice and demonstrate that this is a viable model that offers new insights for the treatment of infertility and understanding the mechanisms of spermatogenesis in domestic animals. However, improvement of the methodologies is necessary in future for completely restoring spermatogenesis *in vitro*.

### 4.3 Generation of 3D testicular culture models

Three-dimensional culture models have gained prominence in research due to their architectural and functional resemblance to native microenvironments. In domestic species, the generation of testicular organoids has become a valuable tool for studying testicular function and development. Notably, testicular organoids have been successfully established from testicular tissues, where cells were harvested using a two-step enzymatic digestion process. These organoids, characterized by an encapsulated shape, contained testis-specific cell types such as germ cells, Sertoli cells, Leydig cells, and peritubular myoid cells.

In large animals, testicular organoids have been generated by isolating testicular cells from bovine testes and culturing them in ultra-low attachment plates with Matrigel (107). These organoids contained Leydig, Sertoli, and peritubular myoid cells, displaying specific localization and changes in number. The developed bovine testicular organoids exhibited steroidogenic activity, characterized by the production of testosterone into the culture media. However, these organoids lacked spermatogonia, limiting the ability to recreate spermatogenesis mechanisms *in vitro*.

Recently, Tang and colleagues (108) successfully established an *in vitro* 3D neonatal testicular organoid culture system containing bovine gonocytes that were cultured for a period of 28 days. Supplementation with GDNF, FGF2, and LIF helped maintain a high proportion of proliferating cells while promoting the transformation of gonocytes into SSCs (108). Additionally, FSH and testosterone were found to be beneficial for maintaining the viability and proliferation of cells in organoids (108). However, these testicular organoids did not exhibit critical testicular compartmentalization, and spermatogenesis was not studied.

Another significant advancement in the field of 3D culture platforms for domestic species is the development of testicular extracellular matrix (ECM)-derived scaffolds through tissue decellularization (109). This approach offers promising avenues for exploring cell-matrix interactions during spermatogenesis. By effectively removing cells and debris while preserving the native ECM composition, 3D structure, and biological activity, these scaffolds create a supportive environment for repopulation with SSCs and somatic cells (110). Tailored decellularization protocol using physical, chemical, or biological agents can be optimized for the unique characteristics of domestic animal tissues, thereby advancing applications such as artificial testis generation, fertility restoration, and drug screening in both large and small animals.

Vermeulen and colleagues were the first to apply and compare several decellularization protocols for prepubertal porcine testicular fragments (111). Following their work, decellularized testes have been obtained in cattle (112, 113) and sheep (114). The derived ECM can be used as 3D scaffolds or lyophilized.

Lyophilization of the decellularized testis allow for the preparation of a hydrogel. Recently, using this approach, porcine testicular organoids have been generated by encapsulating testicular cell suspensions (115). These organoids were maintained for 45 days in culture and consisted of tubule-like structures surrounded by interstitial cells; however, germ cell maturation was not achieved.

In another study, testis-derived scaffolds were fabricated from ram testicular tissue (116). These biological scaffolds were seeded with neonatal mouse testicular cells and supported the formation of organoids that, despite lacking the typical testicular architecture, were able to produce hormones and formed post-meiotic cells (116).

These findings indicate that the generation of 3D culture platform to recreate spermatogenesis *in vitro* is complex and involves well-orchestrated interactions among hormones, growth factors, cytokines, and ECM-derived biochemical and biomechanical cues and presents significant challenges. Additionally, the lack of knowledge regarding the niche microenvironment, nutritional requirements, and the regulatory mechanisms driving self-renewal, proliferation, and differentiation in domestic species has hindered progress in this field. Therefore, it is desirable to develop reliable 3D *in vitro* models that faithfully mimic the architecture and physiological microenvironment of native testicular tissue, bridging the gap between *in vivo* complexity and the oversimplified conventional two-dimensional *in vitro* cultures.

## 5 Future directions and conclusions

Despite significant progress has been made in the cryopreservation and culture of testicular tissue and cells, several challenges remain before these techniques can be translated into reliable veterinary applications. The optimization of cryopreservation protocols requires further refinement to ensure consistent preservation of tissue architecture, cell viability, and functionality across different species and developmental stages. Comparative studies investigating cryoprotectant combinations, fragment size, and warming strategies will be crucial for establishing standardized and effective methods. Concurrently, advancements in testicular tissue and SSCs culture systems must evolve toward more physiologically relevant platforms capable of supporting complete spermatogenesis. The implementation of biomimetic platforms such as testicular organoids, 3D scaffolds and ECM-derived hydrogels represents a promising direction for recreating the native testicular microenvironment. However, the lack of species-specific knowledge on somatic-germ cell interactions, hormonal regulation, and niche dynamics continues to hinder the full maturation of germ cells in culture. The integration of cryopreservation and long-term *in vitro* culture approaches, supported by molecular, epigenetic, and functional analyses, will be essential to assess the safety, efficiency, and reproducibility of these systems. Ultimately, the convergence of optimized cryopreservation and culture protocols could enable the development of species-specific fertility preservation strategies applicable to both domestic and wild animals, with implications for breeding management, biodiversity conservation, and ARTs in veterinary medicine.
